# The impact of age on outcomes of coronary artery bypass grafting

**DOI:** 10.1186/s13019-020-01201-3

**Published:** 2020-07-01

**Authors:** Anthony Lemaire, Cassandra Soto, Lauren Salgueiro, Hirohisa Ikegami, Mark J. Russo, Leonard Y. Lee

**Affiliations:** Division of Cardiothoracic Surgery, Department of Surgery, RUTGERS-Robert Wood Johnson Medical School, 125 Paterson Street, New Brunswick, New Jersey 08903 USA

**Keywords:** Age, Gender, Coronary artery disease, And coronary artery bypass grafting

## Abstract

**Objective:**

As the population ages, increasing number of older patients are undergoing adult cardiac surgery. The purpose of the study is to assess the impact of age on postoperative outcomes in patients that undergo coronary artery bypass grafting (CABG).

**Methods:**

Patients that are ≥70 years old who underwent CABG were selected from the Nationwide/National Inpatient Sample from 2010 to 2015 using ICD-9-CM diagnosis and procedure codes. The patients who were 70–79 years old were compared to patients aged 80–89 years old to determine if the age difference of the patients had an impact on surgical outcomes. In addition, a secondary endpoint is to compare surgical outcomes between the 2 genders of the patients 80–89 years old. The rates of postoperative complications, and mortality were compared.

**Results:**

A total of 67,568 patients were identified who were ≥ 70 years old and underwent CABG. Compared to the Septuagenarians, the Octogenarians were more likely to develop cardiac complications (OR [odds ratio] =1.20, 95% CI [confidence interval] 1.12–1.23. They were also more likely to develop renal complications (*P* < 0001), and respiratory complications (P < 0001). The Octogenarians were also more likely to bleed postoperatively (*P* < 0.0001) and have a higher mortality (*P* < 0001). Furthermore, the female Octogenarians had a higher mortality (OR 1.25 95% CI 1.07–1.46) compared to males in the same age group.

**Conclusions:**

The patients who were ≥ 80–89 years old had worse postoperative outcomes. The Octogenarians who were females had a higher mortality compared to their male counterparts.

## Introduction

**Fig. 1:**
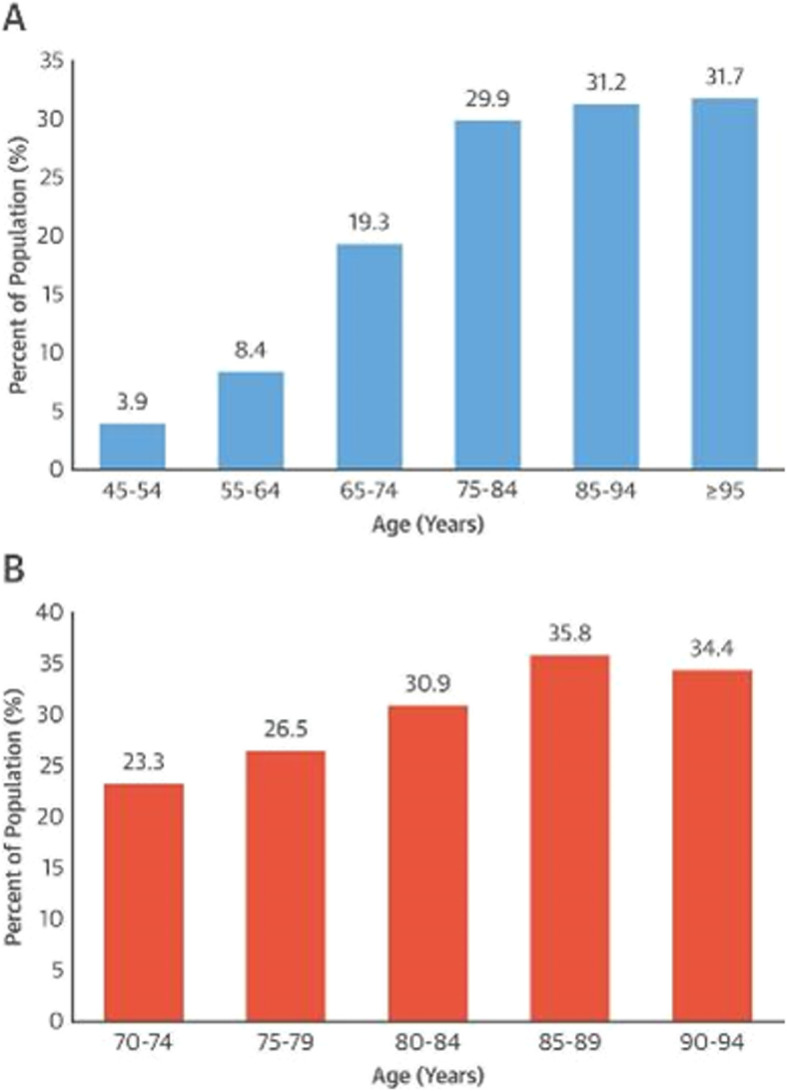
The correlation of age and incidence of coronary artery disease in Women (Red) and Men (Blue)

As the population ages, an increasing number of older patients are being referred for coronary artery bypass grafting (CABG) for cardiovascular diseases [1, 2]. Octogenarians, as the fastest growing stratum of the population and with the highest prevalence of coronary artery disease, are particularly more often being sent to cardiothoracic surgeons for surgical revascularization (Fig. 1) [3]. Similarly, in Germany, during the years 1989 to 2000, there was an increase in the proportion of patients aged ≥70 who underwent cardiac surgery from 11.2 to 36.7% [4]. One of the concerns of operating on older patients is often the trepidation of poor surgical outcomes. There is an apprehension that patients at an advanced age will not recover as well or perhaps have fragile tissue. The same alarm is shared by cardiologist who consider Octogenarians at the highest risk for procedural complications during percutaneous coronary interventions (PCI) owing to their greater prevalence of associated comorbidities and more depressed cardiac function [5, 6]. Although, the results of CABG among Octogenarians are inferior to those of younger patients, CABG outcomes for Octogenarians are better than that of PCI or medical therapy alone [7].. Currently, there is no clear consensus in the literature on the impact of age on CABG patient outcomes.

The purpose of the study is to assess the impact of age on postoperative outcomes in older patients that undergo CABG. Specifically, the aim is to determine whether Septuagenarians have better results compared to Octogenarians and assess the effect of gender in octogenarians on postsurgical outcomes.

## Methods

The data for this project was obtained from the AHRQ (Agency for Healthcare Research and Quality) HCUP (Healthcare Cost and Utilization Project) Nationwide/National Inpatient Sample (NIS) for the years 2010–2015. This is the largest all-payer database in the United States covering about 20% of all hospitalizations in the acute care community hospitals that is up to 8 million for each year. The detailed information about the elements of the NIS database is available at https://www.hcup-us.ahrq.gov/db/nation/nis/nisdbdocumentation.jsp.

The study population included patients aged ≥70 years old who were hospitalized for coronary artery disease (CAD) and underwent CABG during seven days after hospitalization. To select these patients, we used the ICD-9-CM (International Classification of Diseases, Ninth Revision, and Clinical Modification) diagnosis codes 41,001, 41,011, 41,021, 41,031, 41,041, 41,051, 41,061, 41,071, 41,081, 41,091. The ICD code for CAD is 41,401, and CCS code for CABG is 44. The ICD and CCS code was used for the principal diagnosis to limit the sample to patients aged 70–89 years old who were electively (ELECTIVE = 1) or non-electively (ELECTIVE = 0) hospitalized for MI (initial episode) or CAD (principal diagnosis for both) and underwent CABG (any procedure position) during 7 days after hospitalization.

Demographic information was collected for each patient. Pre-, intra-, and post-operative findings were identified through a thorough review of the data. Postoperative complications, morbidity, and survival information were also reviewed. Patients that are ≥70 years who underwent CABG were selected from the Nationwide/National Inpatient Sample from 2010 to 2015 using ICD-9-CM diagnosis and procedure codes. The patients who were ≥ 70–79 years old were compared to patients aged ≥80–89 years old to determine if the age difference of the patients had an impact on surgical outcomes. A secondary endpoint, is to determine if the gender of the octogenarians influenced surgical outcomes. The rates of postoperative complications, mortality, hospital length of stay (LOS) and cost were compared using the Chi-square test, multivariable logistic regression analysis, and Wilcoxon rank sum test.

## Results

**Fig. 2:**
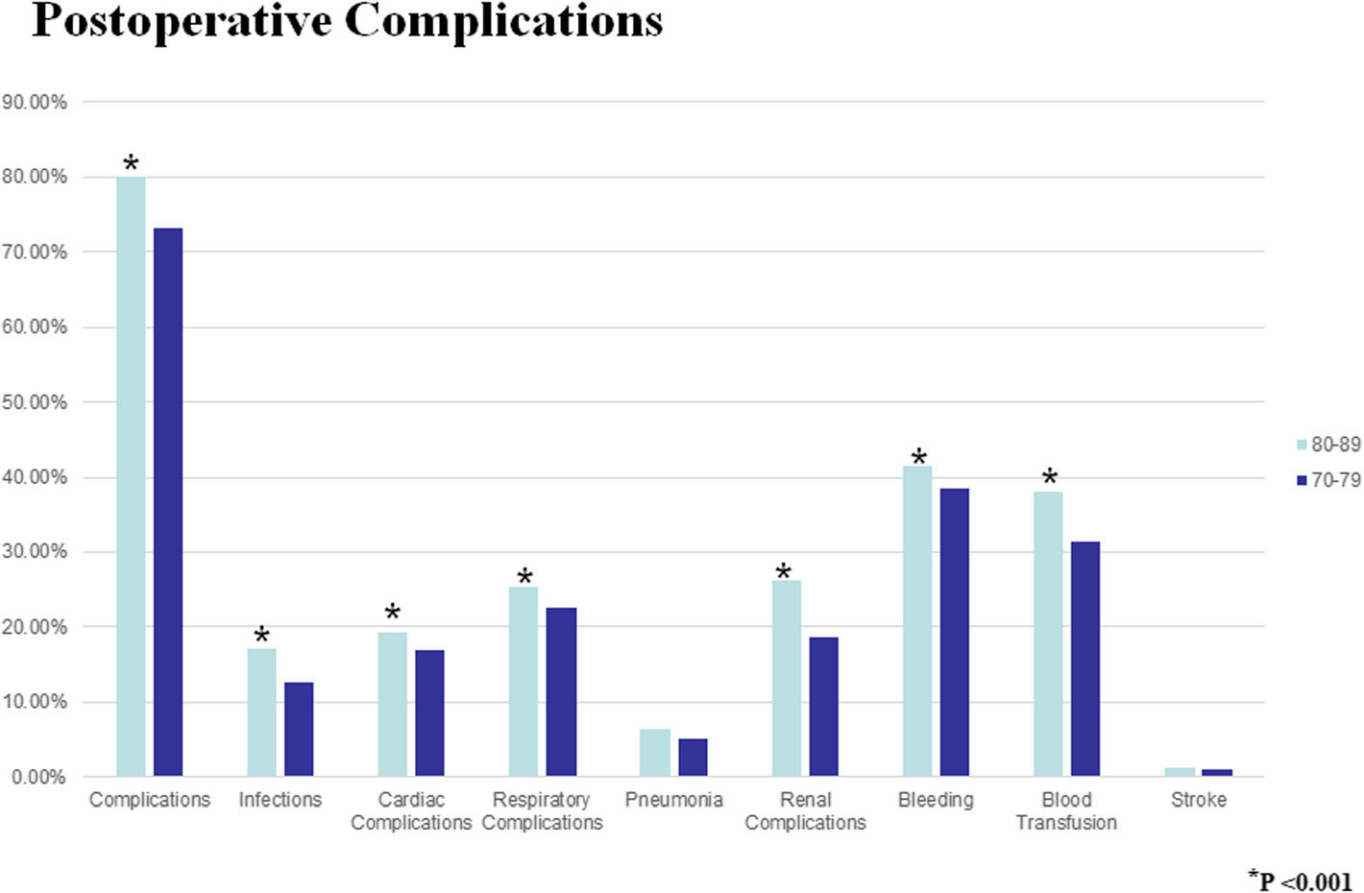
A comparison of postoperation complications after Coronary artery bypass grafting in Septuagenarians and Octogenarians

A total of 67,568 patients were identified who were ≥ 70 years old and older and underwent CABG. The data from our study shows that patients who were octogenarians had worse results (Fig. 2). Specifically, compared to the Septuagenarians, the Octogenarians were more likely to develop more cardiac complications (OR [odds ratio] =1.20, 95% CI [confidence interval] 1.12–1.23. They were also more likely to develop more renal complications (OR 1.54 95% CI 1.48–1.61, *P* < 0001), respiratory complications (OR 1.2, 95% CI 1.2–2.1, P < 0001), and infectious complications (OR = 1.41, 95% CI 1.34–1.48, P < 0001). These complications lead to poor surgical outcomes. Our results are supported by previous studies with similar findings that older patients have worse results compared to younger patients. The unique study design was to separate elderly patients into Septuagenarians and Octogenarians allowing for the identification of important differences in outcomes.

In a study analyzing 8769 patients undergoing cardiac surgery, patients over the age of 70 were observed to have increased risk of postoperative complications including pneumonia, arrhythmia, and bleeding [4]. Similarly, a study published in 2011 demonstrated that risk factors for reoperation due to bleeding were related to older age [8]. As older patients undergo CABG, it is very useful to be able to risk stratify these patients and identify which age group above 70 years old have better outcomes and should be directed to CABG. Conversely, identifying which patients have worse outcomes can help steer these patients towards PCI.

**Fig. 3:**
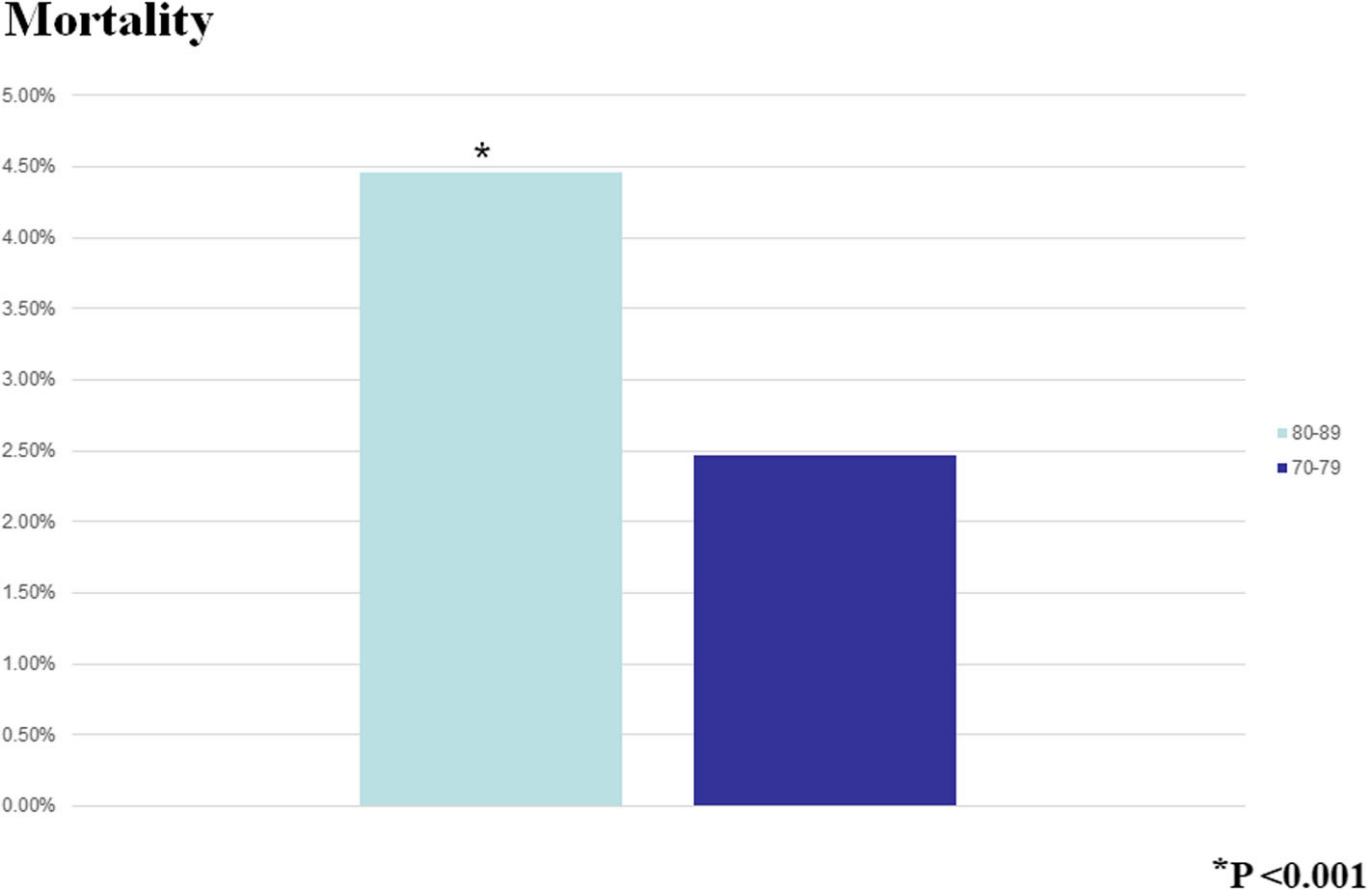
A comparison of mortality after coronary artery bypass grafting in Septuagenarians and Octogenarians

**Fig. 4:**
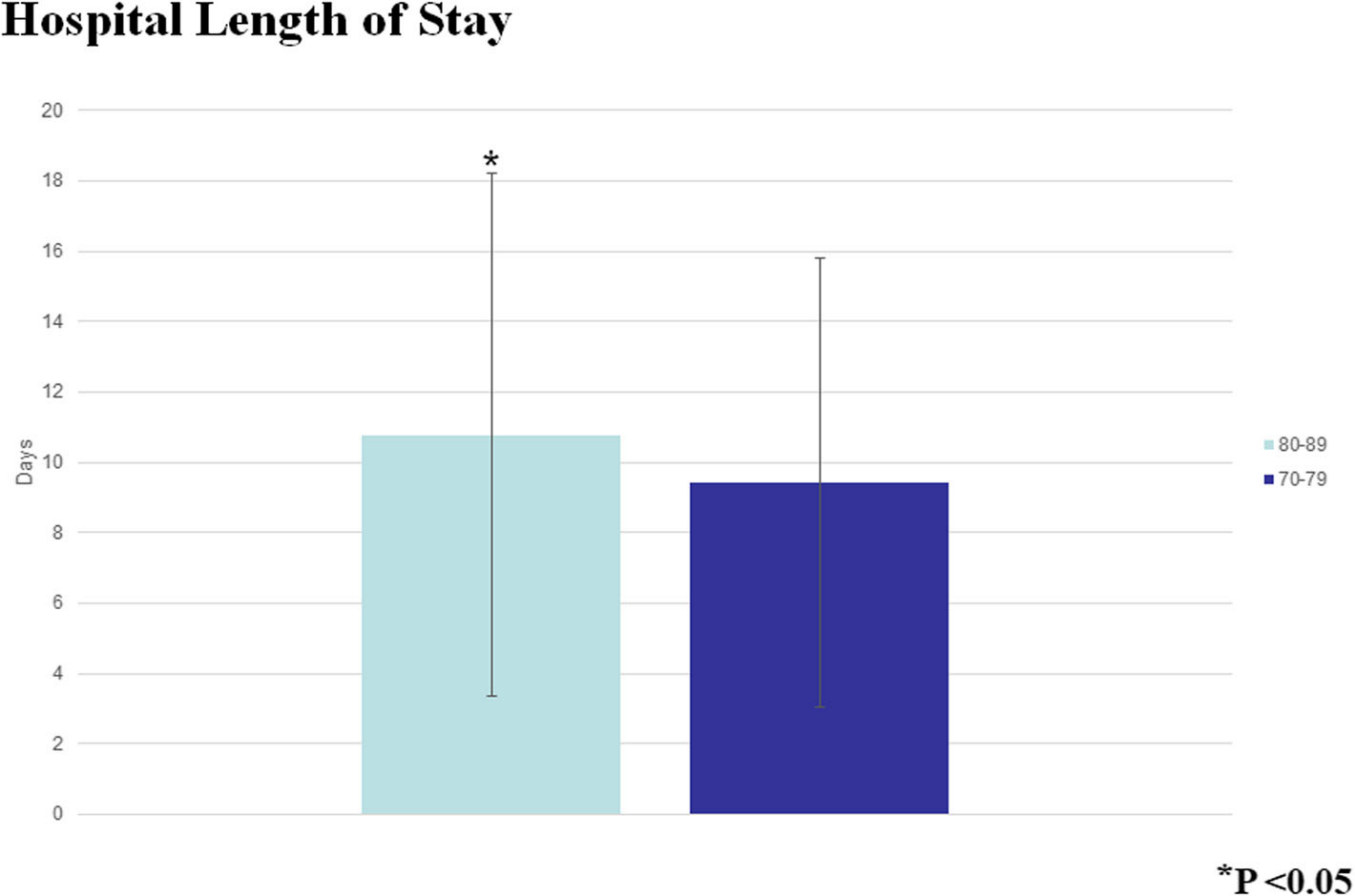
A comparison of hospital length of stay after coronary artery bypass grafting in Septuagenarians and Octogenarians

**Fig. 5:**
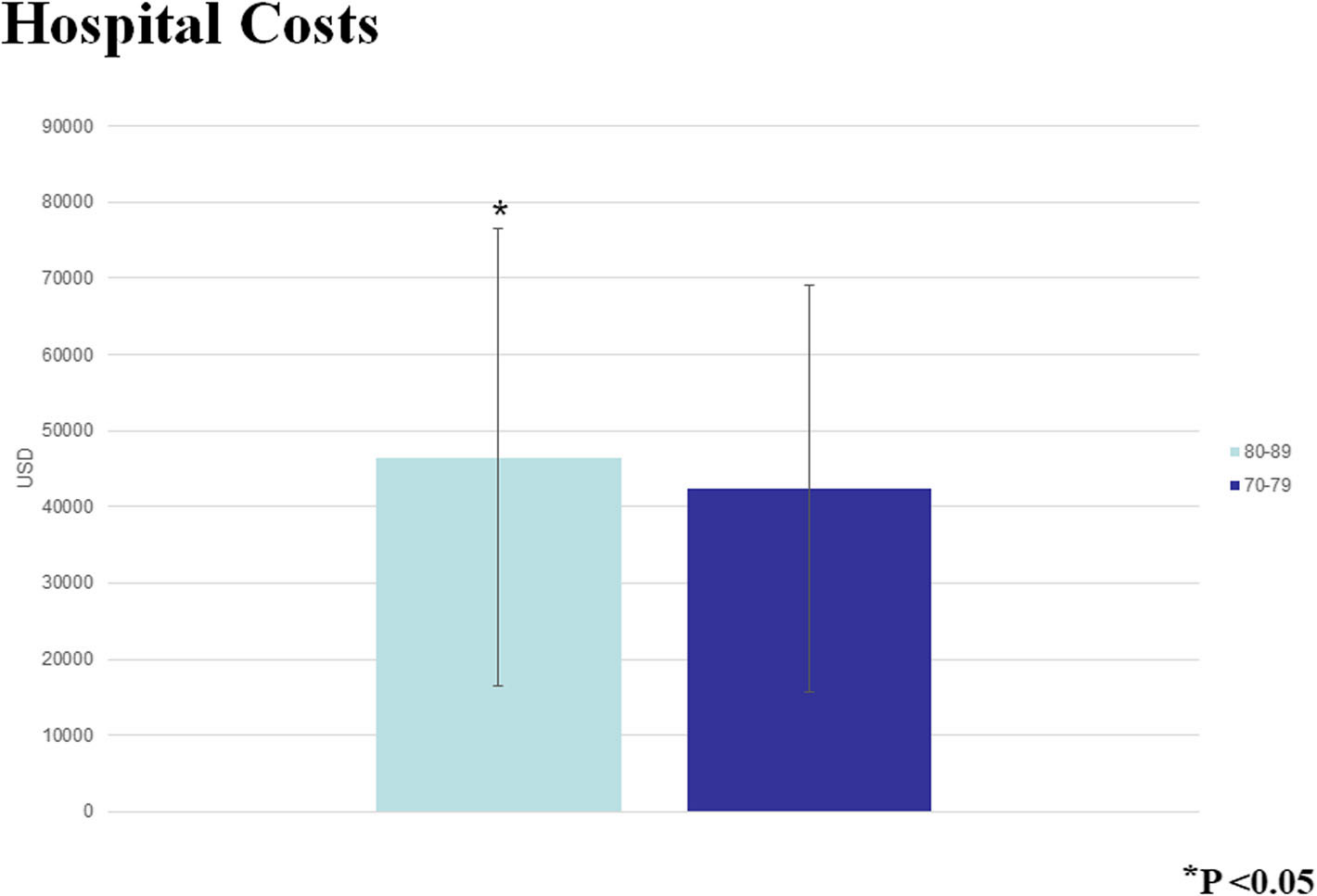
A comparison of hospital costs after coronary artery bypass grafting in Septuagenarians and Octogenarians

**Fig. 6:**
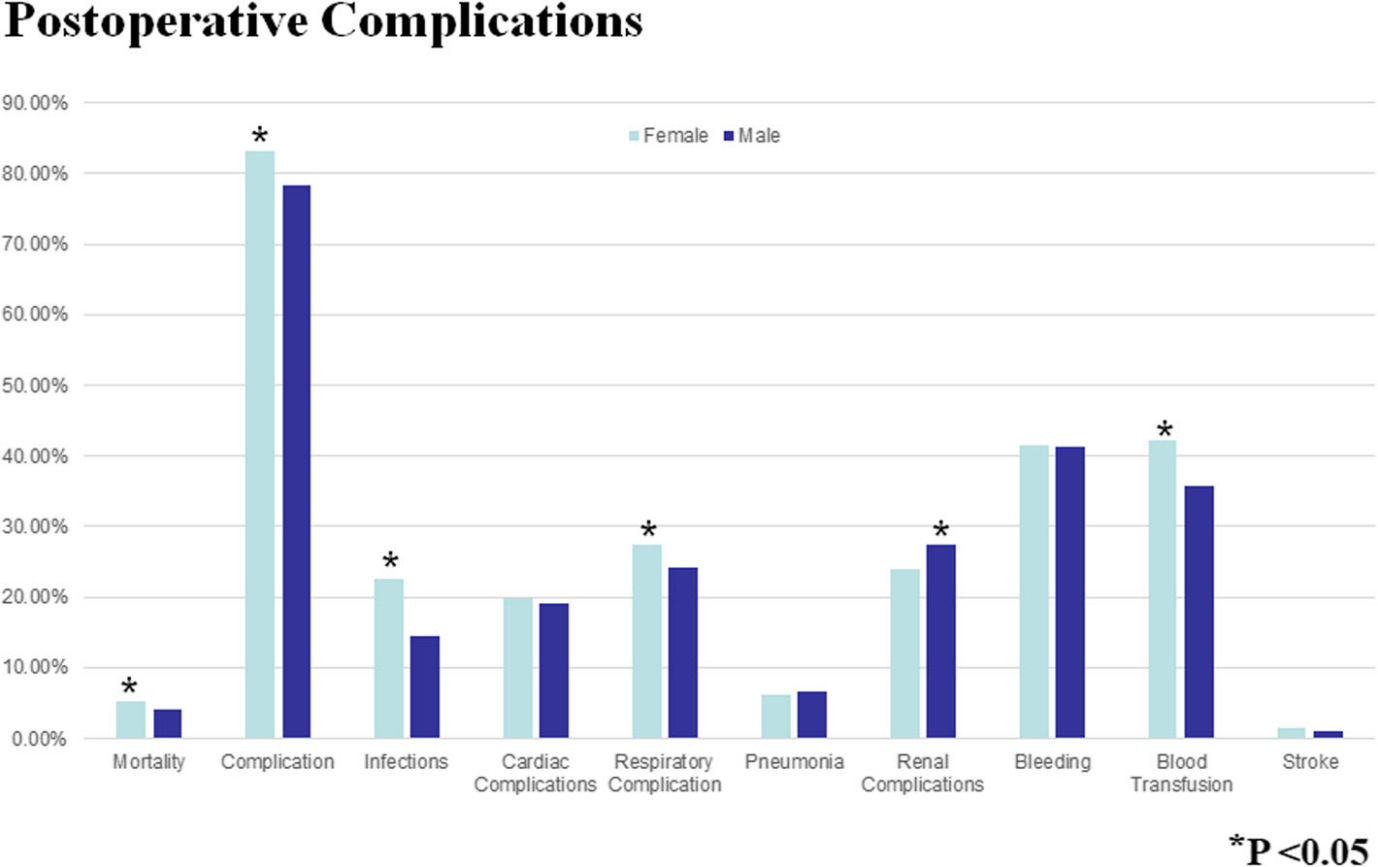
A comparison of postoperative complications after coronary artery bypass grafting in Octogenarians based on gender

**Fig. 7:**
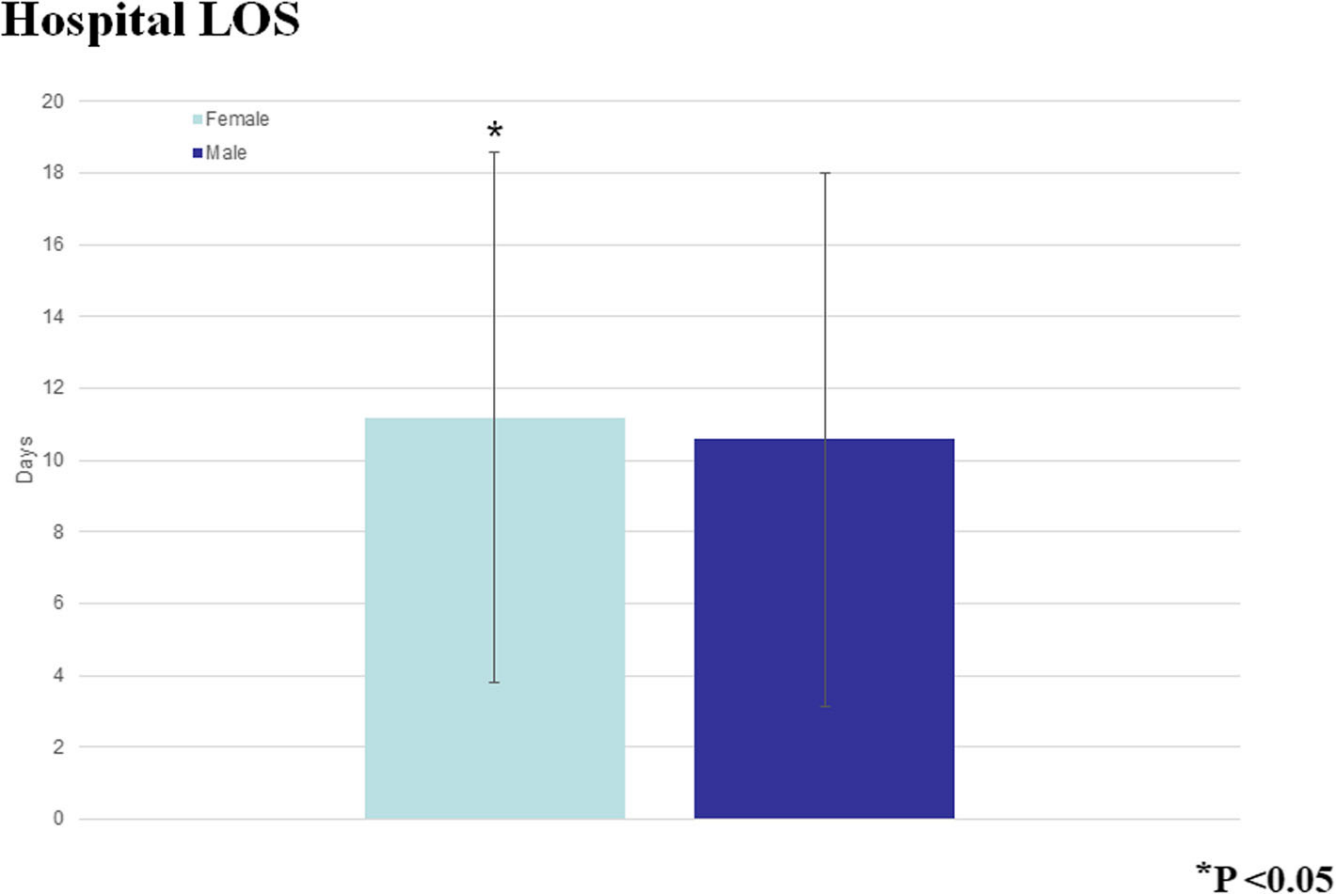
A comparison of hospital length of stay after coronary artery bypass grafting in Octogenarians based on gender

**Fig. 8:**
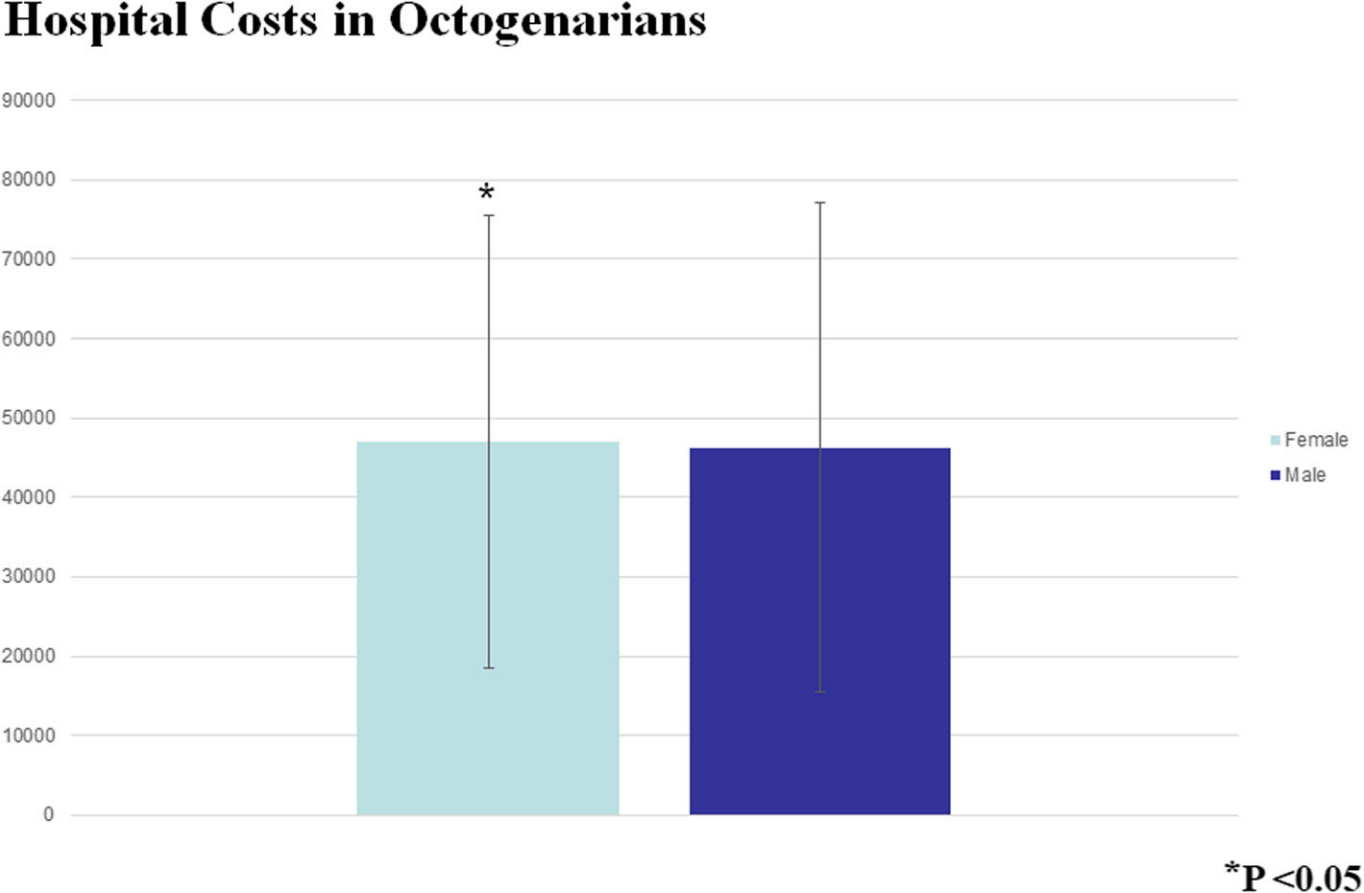
A comparison of hospital costs after coronary artery bypass grafting in Octogenarians based on gender

The Octogenarians in our study were also more likely to bleed postoperatively (*P* < 0.0001), and they had a higher mortality (OR 1.41 95% CI 1.36–1.61, *P* < 0001) (Fig. 3). Moreover, the older patients had a longer postoperative LOS (median 9 days IQR [interquartile range] 7–13 days compared to the Septuagenarians (median 8 days IQR 6–11 days). As expected with the longer LOS, the Octogenarians had an associated increased hospital cost (median $39,152 IQR $30, 0003.84 - $53, 272.84) compared to the Septuagenarians (median $35,996.16 IQR $27,735.94 - $48,134.38) (Figs. 4 and 5). Furthermore, the female octogenarians had a higher mortality (OR 1.25 95% CI 1.07–1.46) compared to males in the same age group (Fig. 1). The gender difference in the Octogenarians is a critical finding not previously reported in the literature. These worse outcomes for female Octogenarians extended to more postoperative complications including bleeding, respiratory complications, and infections (Figs. 6, 7 and 8).

## Discussion

The findings of the study show that Septuagenarians have better surgical outcomes compared to Octogenarians undergoing CABG, with the latter group exhibiting worse results in females. Specifically, the Septuagenarians overall had less complications and mortality than the Octogenarians. The impact of these results allows cardiothoracic surgeons to advise medical professionals that patients above 80 years old should have percutaneous revascularization if the option is available. The design of the study is unique in that most of the studies in the literature compare older patients to younger patients with a wider age range.

In patients undergoing CABG, age and gender are independent risk factors for morbidity and mortality [9, 10]. However, some studies have argued that preoperative risk factors and treatment methods are responsible for the perceived effects of age and sex [11] and others have reported no significant differences attributable to these factors [12]. In 2012, Nicolini et al. [13] investigated early and late outcomes in octogenarians undergoing CABG, advocating that advanced age should not be a deterrent for CABG in carefully selected patients. They showed that candidate selection based on evaluation of systemic comorbidities offered the greatest benefit to successful revascularization. These findings, although different from our study results, highlight the fact that careful patient selection, regardless of age, is critical in surgical outcomes.

Further support of our results comes from Nicolini et al. in a follow up study to his previous work, in which they determined that patients’ ≥80 years old had the highest of all cause and cardiac related death, as well as, increased rates of re-hospitalization and repeat revascularization with PCI [14]. Additionally, Piatek et al. [15] reported a mortality of 7% in octogenarians compared to 3.4% for all CABG procedures at their institution. Prolonged mechanical ventilation, thoracotomy, and longer duration of procedure are described as risk factors for in-hospital mortality in this group, while higher LVEF (Left Ventricular Ejection Fraction) and LIMA (Left Internal Mammary Artery) graft implantation were found to decrease in-hospital mortality. In contrast, Smith et al. [12], reported that CABG in Octogenarians is as safe as and no costlier than in Septuagenarians. However, the relatively small number of Octogenarians (*n* = 71) compared to young (*n* = 579) and old (*n* = 384) Septuagenarians limit the impact of this study.

An additional aspect evaluated in our analysis is the gender difference on outcomes in Octogenarians. There is a perception amongst cardiothoracic surgeons that elderly women have worse surgical outcomes than men do. The premise is based on the thought that older women are frailer and as a result not as robust to handle open-heart surgery. In fact, one of the significant benefits of transcatheter aortic valve replacement (TAVR) is that a median sternotomy is avoided in “elderly” and “frail” patients. This luxury is not afforded for cardiothoracic surgeons who generally must perform a median sternotomy to perform CABG. As a result, being able to decipher which patients may benefit from CABG over PCI is critical to generating optimal outcomes. The findings of the study are supported in the literature [16–18]. Furthermore, most reports in CABG suggest that female gender is an incremental risk factor for adverse outcome [19].

In an assessment of CABG in 1303 patients, Miskowiec et al. [10] reported females undergoing CABG were significantly older (67.3 vs. 62.8 years, *p* < 0.001) than males and were subject to higher 30-day mortality (7.6% vs. 2.8% p < 0.001). Based on their analysis, they determined that female sex was an independent risk factor for death after isolated CABG, which supports our findings of higher mortality (OR 1.25 95% CI 1.07–1.46) in females compared to males. Our analysis also revealed significantly higher infections in females (OR 1.7206 95% CI 1.58–1.87). The higher infections in females were also reported by Al-Alao et al. [11], however, they also reported that early outcomes in females were similar to their matched males. Koch et al. [20] additionally reported that in matched patients, female sex was not associated with increased mortality after CABG.

Furthermore, Bernt et al. [21] reported no significant difference in complications and major morbidity between males and females, suggesting that gender disparities in outcomes maybe improved through individual revascularization strategies. Bukkapatnam et al. [22] evaluated the operative mortality in a large cohort undergoing isolated CABG and determined that operative mortality was significantly higher in females than in males (4.60% vs. 2.53%, *p* < 0.0001). They also found that females were less likely to receive an internal mammary artery (IMA) graft. Leavitt et al. [23] and Piatek et al. [15] reported left internal mammary artery graft implantation decreased mortality, supporting Bukkapatnam et al.’s [22] interpretation that decreased IMA use contributed to the higher mortality in females. As there are numerous hormonal and sex specific variables that may affect coronary vessel disease and CABG outcomes, this is an area of study that requires further in-depth analysis.

## Conclusion

Finally, the study findings demonstrates that advanced age impacts surgical outcomes after CABG with octogenarians having worse postoperative outcomes including higher complications and mortality than septuagenarians. Additionally, in octogenarians, females had a higher mortality than their male counterparts did. An explanation for the worse outcome in the female group is most likely multifactorial and requires additional explanation. Taken together, our results demonstrate that a careful assessment of older patients must take place to determine the best management strategy to provide coronary revascularization.

## Study limitations

The retrospective design as well as the issues with large database studies limits the impact of our findings. As IMA usage data was not collected for our present study, we cannot establish the impact of IMA grafting on the increased female mortality in octogenarians. Additionally, the exclusion of patients undergoing combined valve and CABG procedures may limit the application of our conclusions to these patients.

## Data Availability

The data is available upon request.

## Abbreviations

CABG: Coronary Artery Bypass Grafting
PCI: Percutaneous coronary intervention
AHRQ: Agency for Healthcare Research and Quality
HCUP: Healthcare Cost and Utilization Project
NIS: Nationwide Inpatient Sample
CAD: Coronary artery disease
TAVR: Transcatheter Aortic Valve Replacement
